# Nicotine in Senescence and Atherosclerosis

**DOI:** 10.3390/cells9041035

**Published:** 2020-04-22

**Authors:** Ann Marie Centner, Pradeep G. Bhide, Gloria Salazar

**Affiliations:** 1 Department of Nutrition, Food and Exercise Sciences, College of Human Scinces, 120 Convocation Way, Florida State University, Tallahassee, FL 32306, USA; amc18ek@my.fsu.edu; 2 Department of Biomedical Sciences, FSU College of Medicine, 1115, West Call Street, Tallahassee, FL 32306, USA; Pradeep.bhide@med.fsu.edu; 3 Center for Advancing Exercise and Nutrition Research on Aging (CAENRA), Florida State University, Tallahassee, FL 32306, USA

**Keywords:** cigarette smoke, nicotine, cotinine, senescence, VSMC, atherosclerosis, ApoE^−/−^

## Abstract

Cigarette smoke is a known exacerbator of age-related pathologies, such as cardiovascular disease (CVD), atherosclerosis, and cellular aging (senescence). However, the role of nicotine and its major metabolite cotinine is yet to be elucidated. Considering the growing amount of nicotine-containing aerosol use in recent years, the role of nicotine is a relevant public health concern. A number of recent studies and health education sites have focused on nicotine aerosol-induced adverse lung function, and neglected cardiovascular (CV) impairments and diseases. A critical review of the present scientific literature leads to the hypothesis that nicotine mediates the effects of cigarette smoke in the CV system by increasing MAPK signaling, inflammation, and oxidative stress through NADPH oxidase 1 (Nox1), to induce vascular smooth muscle cell (VSMC) senescence. The accumulation of senescent VSMCs in the lesion cap is detrimental as it increases the pathogenesis of atherosclerosis by promoting an unstable plaque phenotype. Therefore, nicotine, and most likely its metabolite cotinine, adversely influence atherosclerosis.

## 1. Introduction

CVD is the leading cause of death in the United States (USA) and worldwide. The 2019 report of heart disease and stroke statistics from the American Heart Association reported that poor lifestyle behaviors and lifestyle-related risk factors are the foremost causes of death and disability due to CVD [1]. Among lifestyle risk factors, smoking accounts for one third of all deaths from CVD, with a total of 7.1 million deaths worldwide due to cigarette smoke in 2016 [1]. It is estimated that male and female smokers die 12 and 11 years earlier, respectively, compared with non-smokers. In addition to poor lifestyle choices, aging is considered the major non-modifiable risk factor in the development of CVD [2]. Therefore, the added detrimental effect of smoking puts older adults at a higher risk of disease development.

Cellular senescence, which is a hallmark of mammalian aging, is a process in which cells stop proliferating and become dysfunctional due to an accumulation of mutations that cause DNA damage. The reduction in proliferating cells over time impairs repair mechanisms, which are needed to cope with normal wear and tear [3]. Carcinogens present in tobacco, as well as radiation and chemotherapy used in cancer treatment, cause DNA damage that accelerates senescence [4] and may contribute to the increased incidence of CVD in smokers. In addition to cell cycle arrest, senescent cells secrete an abnormal variety of molecules, including inflammatory cytokines, growth factors, reactive oxygen species (ROS), and extracellular matrix components that modify the cellular microenvironment, creating a vicious cycle of inflammation and oxidative stress that causes tissue dysfunction during aging. This process is known as the senescence-associated secretory phenotype (SASP) [5]. While senescence protects against the initiation of tumorigenesis due to a lack of proliferation, the SASP promotes the proliferation of an established tumor [6]. SASP components such as ROS promote senescence in bystander cells, which contribute to the spread of senescence in tissues during aging. Therefore, senescent cells are considered a common target in therapeutic interventions against age-related diseases such as CVD and cancer [3].

This review focuses on tobacco and nicotine in the context of cellular senescence and atherosclerosis. Considering the rise of vaping nicotine aerosols and increased mortality related to vaping, the contribution of nicotine and its major metabolites to CVD is an urgent public health issue. This review also discusses variations in nicotine metabolism and clearance to highlight differences between genders, races, and disease states, all of which play a role in the damage incurred with nicotine use and may be useful for targeted interventions. Animal models of tobacco smoke and nicotine exposure, as well as those of atherosclerosis, are described, and major findings are highlighted. Relevant cell models and cell signaling are also discussed, with an emphasis on the effects of nicotine and smoking in modulating the function of VSMCs, which are the most abundant cells in the vasculature. Although evidence is limited, both tobacco smoke and nicotine appear to induce a phenotypic switch in VSMCs [7,8], inducing migration and proliferation into the intima, or inner layer of the artery. VSMCs play a crucial role in atherosclerosis by forming a new layer called the neointima, which eventually becomes an atherosclerotic plaque through immune cell recruitment and lipid and cholesterol infiltration and accumulation. VSMCs greatly influence plaque stability. In a developing plaque, VSMCs secrete collagen and other components of the extracellular matrix (ECM) to stabilize the plaque [9]. However, in a more advanced plaque, VSMCs can become senescent and reduce plaque stability through the SASP [10], which involves ECM destabilizing protease and inflammatory molecule secretions. Finally, a connection between nicotine-exacerbated atherosclerosis and the acceleration of VSMC senescence is discussed. This leads to the hypothesis that nicotine, like the potent vasoconstrictor Angiotensin II (Ang II), activates p38MAPK and ERK signaling, and upregulates inflammation and the ROS-producing Nox1 in VSMCs, to induce cellular senescence and promote an unstable atherosclerotic plaque.

## 2. Tobacco Use

Over a billion people smoke worldwide, and approximately 34 million Americans aged 18 and older smoke, causing more than 480,000 deaths in the USA each year [11]. The smoking population equates to about 13.7% and represents a downward trend, compared with 2015 (20.9%). While smoking has decreased, vaping has become a more recent trend used by approximately 8 million Americans. Younger nicotine users are more likely to vape than smoke. Only 7.8% of younger adults age 18–24 smoke, compared with about 16% of adults age 25–64 [12]. In terms of gender, 15.6% of smokers are men, compared with 12% of women [12]. Smoking is now banned or isolated to a “smoking only” section in most public places, yet, remarkably, 41,000 deaths still occur in the USA from secondhand smoke [13]. The consequences of secondhand vaping, however, are unknown. The new federal law, Tobacco 21, signed into law in December of 2019, raised the age required to purchase tobacco to 21 and The National Academy of Medicine estimates that it will save 223,000 lives among those born between 2000 and 2019 [14].

Smoking is the top modifiable risk factor for CVD, which is the leading cause of death in America, affecting millions each year [15]. Smoking also leads to chronic obstructive pulmonary disease (COPD) and a number of cancers, including lung cancer—the most deadly form of cancer in the USA [11,16]. The large disease burden associated with smoking contributes to a very substantial financial drain, costing the USA over $300 billion annually for direct medical costs and lost productivity. While taxation is heavy on tobacco products—an additional 10% raise is estimated to reduce cigarette usage by approximately 4% in the USA—the impact of the tax increase varies by population. In fact, in adolescents and young adults, heavier taxation is estimated to cut usage by 10% on average [17].

Demographics, including the geographic location, socioeconomics, and education level, play a role in the smoking status. There is a higher proportion of smokers in the Midwest and South compared to other parts of the USA. People earning a lower income, as well as people with a lower education, are more likely to smoke and more adults and minorities smoke compared to other ages and racial groups [12].

Socioeconomics and the level of education also influence one’s likelihood to quit smoking. Quitting smoking is very difficult due to its highly addictive nature and studies estimate that smokers may try an average of 30 times [18]. While approximately two-thirds of Americans claim that they have a desire to quit smoking, only around 7.5% are successful [19]. An income level below $35,000/year and lower education level (below a bachelor’s degree) correlate with a lower likelihood of smoking cessation. Lastly, minorities are less likely to quit smoking [20]. Nicotine replacement therapy (NRT), buproprion, and varenicline are useful tools that can aid in smoking cessation [21,22]. However, the role these aids play in cessation in different populations needs to be assessed, as precision or personalized medicine based on individual biological factors, including genetics, becomes more popular [23]. In addition, with the already high nicotine and tobacco product-associated disease burden contributing to CVD, cancers, and COPD, therapies to better treat these conditions are much needed.

### 2.1. Tobacco and Nicotine

The cigarette is a very efficient drug delivery system, providing a bolus of nicotine to the brain. When a cigarette is burned, over 7000 chemicals are released, over 70 of which are associated with cancer. As the user puffs, nicotine is titrated and absorbed into the mucosal lining of the nose, lips, and mouth. Nicotine travels to the lungs, where alveolar absorption occurs, and the drug travels in the blood through the circulation, reaching the brain in 10–12 s [24]. In the mesolimbic region of the brain, nicotine binds to nicotinic acetylcholinergic receptors (nAchRs) in the ventral tegmental area, stimulating the release of dopamine in the nucleus accumbens [25]. There are 17 different nAchR types, including a combination of five of the subunits—α1-10, β1-4, γ, δ, and ε. Agonists for these receptors include acetylcholine and nicotine [26]. The two nAchRs with a binding affinity for nicotine are (α4)2(β2)3 and (α7)5 [27]. Dopamine release is considered the prime driver of nicotine addiction, although other chemicals are also associated with addiction, and both environmental cues and stressors play a role in addiction [28]. Nicotine also causes the release of neurotransmitters, including norepinephrine and serotonin [29]. Nicotine stimulates the sympathetic neuron through binding to a nAchR, leading to the ion exchange of Na^+^ and Ca^2+^ and triggering norepinephrine release. Norepinephrine travels from the sympathetic neurons to the muscle inducing Ca^2+^ influx, as well as Ca^2+^ release to and from the muscle, respectively. Transmembrane β1 and β2 adrenergic receptors mediate these processes, ultimately resulting in muscle contraction [30].

Nicotine is implicated in an increasing blood pressure (BP) and heart rate, acting on both branches of the autonomic nervous system and promoting both a “fight or flight” response through the sympathetic arm and “rest and digest” response through the parasympathetic arm. Dopamine is associated with a ‘feel good’ sensation and serotonin is associated with a feeling of relaxation and happiness, while norepinephrine increases BP and heart rate and also improves attention, neurodegenerative disorders such as Alzheimer’s and Parkinson’s [31,32], and depression [33]. The smoking cessation drug bupropion is an anti-depressant drug [21], as it prevents the re-uptake of dopamine and noradrenaline. Varenicline is arguably the best smoking cessation aid on the market and has fewer side effects than bupropion [34,35]. Varenicline prevents nicotine from binding to nAchRs, yet is a partial agonist, allowing approximately one-third of dopamine to be released [22]. Drugs used in smoking cessation allow the user to break addiction because smoking no longer correlates with the “rush” produced by dopamine. Overall, therapeutic interventions that take into consideration the reasons for smoking have higher treatment adherence and smoking cessation rates [28].

After smoking and combustion, filtration, and titration, 1.1–1.8 mg of nicotine enters the oral cavity. After 9 min of cigarette smoking, human plasma levels of nicotine increase rapidly to approximately 14 ng/mL within minutes. The plasma concentration ranges from 10 to 50 ng/mL (0.06–0.31 µM), but has been observed to reach 100 ng/mL (0.6 µM) [21]. Arterial blood contains drastically higher—up to ten times higher—nicotine concentrations than venous blood after smoking a cigarette. Regular users roughly smoke a pack of 20 cigarettes a day, corresponding to 22–36 mg of ingested nicotine [24]. Regular smokers reach peak plasma nicotine levels in the afternoon, and these levels wane off until the morning, when they resume smoking [36]. Compared to other nicotine delivery devices, the cigarette alters nicotine plasma levels the quickest. However, similar levels can be achieved with chewing tobacco (7.5 mg) and nicotine gum (4 mg) [36]. After ingestion, these products lead to a peak of almost 15 ng/mL after approximately 30 min, reaching a plateau or slightly declining over 120 min. Chewing tobacco resulted in a very similar maximum concentration, compared to a cigarette, while nicotine gum was over 5 ng/mL lower [36].

In animal models, intravenous, intraperitoneal, and subcutaneous (I.V., I.P., and S.C., respectively) administration represent very efficient nicotine delivery systems. The bioavailability of I.V., I.P., and S.C. is 100% because nicotine is delivered directly to the systemic bloodstream. The bioavailability of nicotine from cigarette inhalation is still very high, at 80–90% [37]. The cigarette is a more efficient delivery system than vaping because vapes have a wide range of bioavailability as there are a number of different systems and vape liquids on the market. In addition, vape products vary in the form of nicotine they use. Therefore, the range of nicotine bioavailability for vaping is approximately 50–60% [38]. Oral routes of administration in humans include lozenges and gum and are less efficient delivery systems as they vary significantly, in part due to how the product is used [39]. The most effective use of these products involves allowing them to sit in the oral cavity so that nicotine can be absorbed in the mucosal membrane rather than swallowed [37]. Animal models utilize nicotine in drinking water, which corresponds to the oral administration of lozenges and gum. The systemic nicotine content produced by nicotine gum is lower than the amount present in the gum because some nicotine is swallowed and undergoes first-pass metabolism (i.e., absorption in the gut and filtration by the liver before systemic distribution). The bioavailability of nicotine for lozenges and gum is approximately 50–80% [39]. Bioavailability of the dosage via oral routes needs to be considered when making claims or designing a study.

Nicotine is a weak base, with a pKa of 8.0. It is 69% ionized and 31% unionized in its free form. Acidity affects the absorption of nicotine. At an acidic pH of 5.5–6.0, nicotine is in its ionized form and is not absorbed by tissues. Nicotine distilled from smoking a cigarette is in an ionized form and does not cross membranes, preventing its buccal absorption. On the other hand, lung alveolar fluid has a pH of 7.4, which converts nicotine into its unionized form, facilitating rapid absorption [39].

Nicotine travels in the blood, and approximately 5% binds to plasma proteins [40], such as albumin; lipoprotein; glycoprotein; and α, β, and γ globulins. There is a trend toward lower serum albumin and significantly higher glycoprotein α_1_-AGP in smokers. α_1_-AGP is an acute phase reaction protein known for its drug-binding properties [41]. Plasma nicotine levels peak near the end of smoking a cigarette and decline quickly over the course of only 20 minutes as the nicotine is disseminated to the body’s tissues. Nicotine is converted into cotinine and other metabolites very quickly. Certain tissues have a higher affinity for nicotine than others. Urakawa et al. studied autopsy samples from human smokers and observed that the nicotine affinity was high in the liver, kidney, spleen, and lung, and was low in the adipose tissue [42]. Nicotine metabolism occurs almost exclusively in the liver. However, in animal models, metabolism can occur in the nasal passage, lung, kidney, and brain [39].

Nicotine metabolism in the liver involves a wide variety of enzymes. The liver enzyme that oxidizes nicotine to cotinine is the cytochrome p450 (CYP) 2A6. Cytochrome p450s are a family of enzymes that play a substantial role in drug and xenobiotic metabolism. To a much lesser degree, another enzyme in this family, CYP2B6, can metabolize nicotine in hepatic tissues and is most active when nicotine concentrations are very high [39].

Cotinine is the major metabolite of nicotine in the human body, as approximately 80% of nicotine is converted into cotinine rapidly—a process that is dependent on the rate of CYP2A6 enzymatic activity [24]. First, nicotine is metabolized by CYP2A6 to a cotinine-iminium ion, which is then converted into cotinine by aldehyde oxidase. Cotinine is largely converted into trans 3’ hydroxy cotinine, which is also catalyzed by CYP2A6. Additionally, nicotine can undergo oxidation by flavin monooxigenase (FMO) and form nicotine N’ oxide, as well as glucuronidation by UDP-glucuronosyltransferase (UGT) to form nicotine glucuronide, although these are minor pathways. In addition to the metabolites mentioned, a number of other minor metabolites, such as glucuronides from cotinine, nicotine, and trans 3’ hydroxy cotinine, are also produced [37].

Hukkanen et al. have summarized early studies of nicotine and cotinine clearance after I.V. administration [39]. After such administration, nicotine has an average half-life of 2 h, while cotinine has a half-life of approximately 16 h in the blood plasma. While nicotine plasma peaks at 30 ng/mL with unrestricted smoking use in humans, cotinine reaches an average of 275 ng/mL. Nicotine is primarily excreted in the urine, although minor amounts of nicotine are lost through sweat and feces. The pH of the system greatly influences excretion, with an acidic pH favoring urinary excretion [43]. Approximately two-thirds of nicotine is converted into cotinine, around 5–6% of nicotine is excreted as nicotine N’-oxide, and 3–5% is excreted as nicotine glucuronide [44,45].

### 2.2. Factors Influencing Nicotine Metabolism

Sex differences exist in nicotine use and metabolism. Females are generally more rapid metabolizers than males. This can lead to more nicotine ingestion (through a larger draw or pull while smoking) in females, compared to males. As suggested by Amiri et al., this leads to an astounding 25% increase in CV pathologies, increasing the risk of first myocardial infarction (MI) and resulting in twice as many years lost due to MI [46,47,48,49,50]. Females are less likely to smoke, but more likely to use cigarettes with menthol—an agent that hastens nicotine clearance and may contribute to heightened addictive behavior [46,51]. There are other agents that accelerate nicotine clearance, such as grapefruit juice [52] and certain drugs, including dexamethasone (used to treat inflammatory conditions) [53] and phenobarbital (an anticonvulsant) [54]. In addition, the consumption of alcohol can accelerate nicotine metabolism by changing enzyme kinetics [55]. In females, in certain instances, CYP2A6 expression or function appears to be upregulated and this may be related to estrogen and progesterone levels [56,57]. Oral contraceptives also contribute to rapid nicotine clearance [58]. Of great consideration is fetal exposure to nicotine via mothers’ nicotine use. It has been observed that neonates have slower nicotine clearance, despite active CYP2A6. This is because, after birth, infants have a reduced hepatic blood flow. Maternal smoking increases the risk of preeclampsia, still birth, and sudden infant death syndrome (SIDS) [59,60].

Pathological conditions can also influence nicotine clearance. A study in rats showed that acute hepatitis or cirrhosis reduces nicotine metabolism due to a decrease in nicotine metabolizing enzyme protein expression [61]. In addition, patients with alcoholic liver cirrhosis who smoke show sustained higher plasma levels of cotinine [62]. CYP enzyme inhibition, as well as hepatic blood flow, influence nicotine clearance; with enzyme inhibition and reduced blood flow corresponding to a slower clearance. Patients with renal disease have slower clearance due to injury-induced reductions in hepatic blood flow. In severe end-stage renal disease, clearance by the liver can be reduced by 50% [63,64].

There are also racially linked variations in nicotine metabolism, which can be largely attributed to different CYP2A6 enzyme alleles. Caucasians are the fastest metabolizers compared to African Americans and Asian ethnicities (Korean, Chinese, and Japanese). Although multiple alleles contribute to individual and racial variability, general ethnic differences in alleles explain slow and fast clearance between races. Caucasians have the highest expression of the very active CYP2A6*1B allele, while a large percentage of African Americans have the less-active allele CYP2A6*17, and Asians have the non-active allelic forms CYP2A6*4 and CYP2A6*9 [65,66,67,68,69].

### 2.3. Tobacco and Nicotine in Animal Models

Murine models are most commonly used for nicotine and tobacco studies [70,71,72,73,74], although other animal models, including primates and *C. elegans*, have been used for behavioral and metabolic studies [75,76,77]. Species differences exist in nicotine metabolism, and non-human primates metabolize nicotine in a similar manner to humans [78]. In humans, while the tissue half-life of nicotine after smoking a cigarette is only about 8 min, the plasma half-life is 2 h. Metabolites of nicotine have a longer half-life. Notably, the half-life of cotinine is 16 h and the half-life of trans 3’ hydroxy cotinine is 7.5 h [24]. In rats, the half-life of nicotine is 45 min, while the half-life in mice is only 6–7 min [79,80]. Mice use CYP2A5, an enzyme ortholog of the human CYP2A6 enzyme, to metabolize nicotine, forming large amounts of cotinine like humans. Rats have inactive CYP2A6 and use CYP1B1/2 to metabolize nicotine, producing similar amounts of cotinine, trans 3’ hydroxy cotinine, and other major nicotine metabolites [24]. Because rat nicotine metabolism differs from human metabolism, in studies in which nicotine metabolites are of importance, the mouse model is preferred. In addition to having a genetic make-up and CYP expression similar to humans, mice are preferred mammalian models because they are small in size and reproduce rapidly. However, the rapid rate at which mice metabolize nicotine must be considered, as clearance is much quicker than in humans. Therefore, drug dosing needs to be adjusted accordingly to maintain plasma levels similar to human levels [37].

When choosing the route of administration in animal models, many factors must be considered, like the purpose of the study (i.e., behavioral or non-behavioral). Behavioral studies benefit most from an efficient delivery system, such as an I.V. injection, because the nicotine bolus quickly travels to the brain to release a host of mood-altering chemicals. However, this method is time-consuming and stressful for both the animal handler and animal. Injection requires the daily (possibly even more than once a day due to rapid metabolism) handling of animals, as well as skill in the technique. In other studies, such as a study more interested in long-term physiological effects, nicotine can be supplied in drinking water. Nicotine in drinking water is less stressful to the animal, as it does not require daily handling or invasive procedures. Nicotine in drinking water mimics the use of chewing tobacco and nicotine gum in humans as nicotine is rapidly absorbed in the mouth. Nicotine in drinking water has been used in a number of recent behavioral studies [70,71,72]. For example, we exposed male C57BL/6 to nicotine (200 µg/mL in drinking water for 12 weeks) and crossed them with female C57BL/6 not exposed to nicotine. The offspring of both genders displayed hyperactivity, attention deficits, and cognitive inflexibility. Offspring were then bred to produce the next generation, which also showed behavioral deficits, but to a lesser degree than their founders. Therefore, behavioral deficits were passed down for two generations. Furthermore, nicotine altered DNA methylation patterns in sperm cells [71]. These data suggest that nicotine may also impose epigenetic modifications in the CV system that could increase the incidence of CVD in future generations. This idea needs to be explored.

Delivery systems in which an osmotic minipump is inserted into the animal to deliver nicotine over extended periods of time are also available [81]. This method is more expensive and is quite invasive, requiring surgery to insert the device under the epidermis. However, it is a remarkable technique that can continuously deliver nicotine using different dosing regimen and time courses. In summary, the nature of the study, monetary and time constraints, stress, and invasiveness must be considered when choosing a nicotine delivery system [37].

There are a number of factors to consider when extrapolating animal study findings to the general human population. Humans are exposed to a wide range of environmental stimuli and have a wide range of habits, preferences, and lifestyles. Understanding the physiological, pathophysiological, and molecular mechanisms underlying nicotine addiction and diseases caused by tobacco and nicotine use can help people overcome their addiction and better treat associated conditions. Statistics—both detailing risks and groups more likely to smoke—and public messaging are important for reaching people and teaching them about the harm of nicotine use, usage risk factors, and nicotine addiction. Taxes can deter people from buying tobacco and nicotine products. Lastly, public policy also greatly influences people’s habits and stricter policy can reduce the use of tobacco and nicotine products and save lives.

As mentioned before, tobacco and nicotine exposure is associated with lung disease [82,83] and cancer [84,85,86]. In this review, we will focus on the effect of tobacco and nicotine in senescence and its contribution to atherosclerosis.

## 3. The Cardiovascular System

Worldwide, CVD results in approximately 17 million annual deaths, half of which are attributed to hypertensive complications [87,88]. Untreated hypertension damages the endothelium, leading to cell proliferation, vascular remodeling, and immune cell recruitment. These processes contribute to pathological blood vessel narrowing and plaque build-up [89]. The vessel diameter is the most important factor in determining blood flow resistance and BP, and is mainly regulated by VSMCs through Ca^2+^-dependent contraction and subsequent relaxation. Smooth muscle is nonstriated and the key event in smooth muscle contraction is the phosphorylation of the myosin light chain (MLC) through MLC kinase (MLCK) activation.

The endothelium can relay signals to the underlying VSMCs via gap junctions. Endothelial cells (ECs) and VSMCs are coupled by myoendothelial gap junctions, which allow a change in membrane potential and intracellular Ca^2+^ to be passed between cells [90]. Gap junctions are formed by a hexamer of connexins (connexon) that interact with a connexon at the adjacent cell, allowing the passage of small molecules and ions [91]. Connexin 43 and connexin 40 are mainly expressed in VSMCs [92] and ECs [93], respectively. In the media, gap junctions also couple one cell cytoplasm to another, allowing electrical signal transduction across the vessel wall and coordinated myogenic responses.

ECs also regulate VSMCs by releasing nitric oxide (NO), through endothelial and inducible NO synthases (eNOS and iNOS, respectively). eNOS is commonly activated by shear stress [94], while iNOS is activated by stress conditions, and is most active during pathological conditions [95]. NO regulates blood flow and vascular tone by two mechanisms. First, by activating guanylate cyclase, NO increases cyclic guanosine monophosphate (cGMP) [96,97,98], leading to the protein kinase G (PKG)-dependent activation of MLC phosphatase. Overall, this pathway reduces MLC phosphorylation and smooth muscle cell contraction. NO also reduces intracellular Ca^2+^ in a cGMP-independent manner by stimulating the reuptake of calcium by the sarco/endoplasmic reticulum calcium ATPase (SERCA) channel in the sarcoplasmic reticulum [99]. Furthermore, NO plays a role in mitochondrial oxygen expenditure by inhibiting cytochrome c oxidase [100].

### 3.1. VSMC Phenotypic Switch

Certain stressors can drastically alter VSMC physiology, inducing a phenotypic switch. In a normal healthy state, VSMCs are differentiated; express contractile genes, such as the myosin heavy chain (MYHC) and alpha-smooth muscle actin (αSMA); and are quiescent. A phenotypic switch can occur when the cell is exposed to growth factors, such as platelet-derived growth factor (PDGF) [101], hypoxia [102], ROS [103], and the activation of DNA damage pathways like p53 [104]. The cell develops a synthetic phenotype and loses contractile markers and contractility, becomes proliferative and migratory, and upregulates the expression of ECM components, such as collagen [105]. The phenotypic switch of VSMCs drives a number of CV pathologies, such as hypertension, atherosclerosis, restenosis (post angioplasty), and aortic aneurysm [106]. In vivo, the phenotypic switch develops in the early stages of atherosclerosis in response the pro-atherogenic conditions, such as PDGF [107].

The contractile phenotype of VSMC is regulated by the master regulator myocardin (MYOCD). MYOCD associates with serum response factor (SRF) on the consensus sequence CC[A/T]6GG, known as the CArG box, in promoter regions leading to the transcription of VSMC contractile genes, such as α-SMA and MYHC. A G/C rich region on the promoter upstream of the CArG box also plays a role in the inhibition of contractile gene transcription. Kruppel-like factor 4 (KLF4) drives the synthetic phenotype by inhibiting MYOCD interaction at the CArG box [108]. MYOCD prevents the phenotypic switch by several mechanisms. For example, by controlling the expression of cell junction proteins. MYOCD induces the expression of miR-206, which inhibits the expression of connexin 43 [109]—a connexin that regulates VSMC proliferation [110]. PDGF signaling is upregulated in VSMCs and drives proliferation in cells undergoing phenotypic switch, and represents one of the most potent regulators of this process. PDGF induces the phosphorylation of connexin 43 by MAPK, leading to an increased interaction of connexin 43 with cyclin E, promoting the progression of the cell cycle [111]. PDGF also promotes KLF4 expression and its binding to promoter regions, suppressing the contractile phenotype. The mechanism involves the activation of stimulating protein 1 (Sp1)—a transcription factor that mediates KLF4 transcription [112]. The synthetic phenotype is further driven by additional signaling pathways, including the Hippo-Yap pathway. This pathway mediates the phosphorylation and inactivation of Yap [113]—a transcription factor that promotes the expression of growth-related genes [114]. PDGF interacts with the Hippo-Yap pathway by increasing Yap expression, which interferes with the formation of the SRF/MYOCD complex and its interaction with the CArG box, and by promoting the expression of cell cycle regulators like Cyclin D1 [115].

MYOCD expression and the VSMC phenotype can be influenced by pro-inflammatory molecules and cholesterol. Inflammatory cytokines, such as interleukin (IL)-1β, promote the synthetic phenotype by downregulating MYOCD in an nuclear factor kappa B (NF-κB) dependent fashion [116]. The microRNAs miR-143/miR-145 are upregulated by MYOCD and target KLF4 to maintain the contractile phenotype. Interestingly, cholesterol downregulates miR-143/miR-145, resulting in VSMC conversion to a macrophage-like phenotype [117,118]. Furthermore, miR-145 was reduced in atherosclerotic lesions in human patients, which correlated with increased KLF5 and reduced contractile gene expression [119]. Transforming growth factor-β1 (TGF-β) and bone morphogenetic protein 4 (BMP4) promote the contractile phenotype by upregulating miR-143/145, which prevent KLF4 expression [120].

An additional pathway leading to a VSMC phenotypic switch and VSMC proliferation is the WNT/β-catenin signaling cascade, which is induced by hyperlipidemia [121]. Specially, WNT3A can cause a phenotypic switch, observed by increased collagen I and III mRNA and matrix metalloproteinase (MMP)-2 and MMP-9 expression and increased gap junction communication, evidenced by high levels of the gap junction protein connexin 43 [122].

### 3.2. Nicotine in VSMC Biology and Phenotypic Switch

The role of cigarette smoke and nicotine in the VSMC function and in particular, the phenotypic switch, is limited. In 2013, Starke et al. [7] reported that cigarette smoke extract (CSE) decreased the markers of differentiation SM22, αSMA, and MYHC, while increasing proliferation in cerebral VSMC. These effects were associated with a decreased expression of MYOCD and increased expression of KLF4 and inflammatory markers like NF-κB, TNF-α, and IL-1β, as well as MMP-3 and MMP-9. KLF4 upregulation seems to modulate this effect, since KLF4 siRNA reduced the effect of cigarette smoke in differentiation markers. Importantly, a reduced expression of differentiation markers was also observed in vivo in rat carotid arteries exposed to CSE. Furthermore, Starke et al. demonstrated that CSE promoted the binding of the histone deacetylase HDAC2 to promoter regions of differentiation markers, leading to reduced histone acetylation and overall expression. Increased levels of the repressive mark H3K27me3 were also observed in these promoters. Therefore, the effect of cigarette smoke in VSMCs is mediated, at least in part, by epigenetic modifications.

Although a pioneer study by Thyberg in 1986 [123] showed that nicotine increased proliferation and reduced the myofilament content in aortic rat VSMC (RASMs), it was not until 2014 that Yoshiyama et al. [8] demonstrated that nicotine was able to induce VSMC phenotypic switch in human aortic VSMCs (HASMs). Similar to CSE, nicotine reduced SM22 and MYHC expression and increased ERK and p38MAPK phosphorylation. The effect of nicotine is likely mediated by nAchRs that are known to be expressed in VSMCs. With the exception of α9, VSMCs express all alpha subunits for this receptor [124]. A study in 2014 on HASMs discovered that nicotine acted through nAchRs and G-protein coupled receptors to increase intracellular calcium and induce smooth muscle cell phenotypic switch to a synthetic type. This phenotype was characterized by a reduction of contractile markers (myosin II 11, caldesmon, and SM22) and an increase in synthetic markers (myosin II 10 and β-actin), leading to VSMC migration to the intima. This phenotype also involved the activation of p38MAPK and ERK signaling [8].

The nicotine signaling cascade was also shown to interact with the Ang II pathway, as demonstrated by Li et al. in RASMs. Together, Ang II and nicotine had significant compounding effects on VSMC proliferation, compared with Ang II or nicotine alone. Activation of ERK, signal transducer and activator of transcription (STAT), and p38MAPK were involved in the nicotine-enhanced Ang II mitogenic cellular response [125]. In addition to proliferation, nicotine also increased cell migration in bovine aortic VSMCs treated with nicotine and 10% fetal bovine serum (FBS), but not with nicotine alone. The increased phosphorylation of ERK and p38MAPK seen with nicotine mediated this effect [126]. Additionally, epidermal growth factor receptor (EGFR)-induced ERK activity was involved in vascular endothelial growth factor (VEGF) release by RASMs [127], suggesting that the VSMC phenotypic switch promotes endothelial cell proliferation. Guo et al. also demonstrated that VEGF is secreted by VSMCs during phenotypic switching in response to PDGF [128].

Changes in microRNA expression have also been reported in response to nicotine. For example, Liang et al. [129] reported that increased expression of miR-200b mediated the upregulation of Ras homologous (Rho) GTPase activity and expression of cytoskeleton proteins. This effect on the cytoskeleton is consistent with other studies showing cytoskeleton remodeling in VSMCs treated with nicotine [130,131].

In vivo, balloon injury of the right iliac artery in nicotine-treated (100 μg/mL in drinking water for 3 months) rats induced neointima formation, which was mediated by an ERK and early growth response 1 (Egr-1)-dependent mechanism [132]. Furthermore, histological examination of aortic wall remodeling by nicotine by Rodella et al. [133] indicated a significant upregulation of thrombospondin-1 (TSP-1), TGF-β1, plasminogen activator inhibitor-1 (PAI-1), and CD31 levels in VSMCs in the media in response to 100 μg/mL nicotine in drinking water. TSP-1 is a glycoprotein secreted by VSMCs in response to injury that mediates VSMC proliferation and migration [134] and arterial remodeling [135]. PAI-1, on the other hand, promotes the migration of VSMC by binding to the low-density lipoprotein receptor-related protein (LRP) [136].

In the case of ECs, cigarette smoke was shown to increase oxidative stress by the activation of NADPH oxidase activity in vivo [137]. Rats exposed to the smoke of five cigarettes per day for one week showed reduced acetylcholine-dependent relaxation and increased expression of IL-1β, IL-6, and TNF-α in carotid arteries, which was prevented by apocynin—an NADPH oxidase inhibitor. Increased oxidative stress by CSE was also observed in bovine pulmonary artery ECs (BPAECs) in culture due to increased NADPH oxidase activity [138] and in rat pulmonary microvascular ECs in culture due to increased xanthine oxidase expression and activity [139]. In support of the role of NADPH oxidases in cigarette smoke effects, Mo et al. [140] reported that CSE effects were attenuated in gpx91phox knockout mouse pulmonary microvascular ECs (MPMVEC). These effects included reduced superoxide levels, ERK and p38MAPK phosphorylation, and IL-6 and Egr-1 levels. Furthermore, serum from smokers increased oxidative stress and reduced NO levels in cultured human ECs [141].

For VSMCs, the source of oxidative stress induced by cigarette smoke was identified as Nox1, which was demonstrated using Nox1 inhibitors and Nox1^−/−^ VSMCs. Nox1 was also involved in oxidative stress and apoptosis induced by nicotine in lung epithelial cells [142].

Finally, cotinine, a major nicotine metabolite, was also shown to influence VSMC proliferation —a mechanism that was associated with increased telomerase activity [142]. ECs isolated from porcine common carotid arteries perfused with either nicotine or cotinine show a similar upregulation in VEGF expression [143]. Therefore, nicotine and its metabolite cotinine are able to similarly affect ECs and VSMCs in arteries, suggesting that nicotine may have a long-lasting effect on the vascular function, even after its metabolism. Furthermore, cotinine’s half-life in circulation is longer than that of nicotine, suggesting that cotinine may mediate the effects of nicotine in the CV system.

In summary, in vitro and in vivo studies support the role of nicotine, as the bioactive component in cigarette smoke, in the initiation of proliferation and migration phenotypes associated with the phenotypic switch of VSMCs. This process seems to strongly depend on activation of the MAPK signaling pathway and oxidative stress mediated, at least in part, by Nox1.

## 4. Senescence Overview

Senescence is defined as an irreversible state of cell cycle arrest categorized as telomere-dependent or intrinsic/replicative senescence and telomere-independent or extrinsic/stress-induced senescence. Telomeres cap the ends of chromosomes in post-mitotic cells, preventing chromosome fusion. Telomere shortening depends on the species [144] and they shorten by 50 to 200 base pairs (bp) with each cell division in diploid cells [145,146]. For example, in human fibroblasts, telomeres are shortened by 50 bp [147], while in human lymphocytes, in culture, the rate is 120 bp per cell division [148]. Hayflick and others observed a limit, which later become known as the Hayflick limit, for the number of cell divisions that post-mitotic cells can undergo (around 50–70 divisions) before becoming quiescent [149]. This limit defines the basis of replicative senescence because it involves the shortening of telomeres over time. This type of senescence is central to the ‘telomere attrition’ theory of aging, which states that the shortening of telomeres over time inevitably leads to decline and death.

Stress-induced senescence, on the other hand, occurs when environmental insult, such as radiation and ROS, drive senescence, and can occur when telomeres are still present and functioning properly. The main mediator of this type of senescence is activation of the DNA damage response (DDR) [150] through ataxia-telangiectasia mutated (ATM) kinase [151], which activates the tumor suppressor p53 [152] and cyclin-dependent kinase inhibitor (CDKI) p21 [153]. A host of factors besides the loss of telomeres can lead to senescence and these factors drive the second type of senescence—environmental or extrinsic. Extrinsic/environmental insults include oncogenic or mitogenic stimuli, DNA damage (radiation, chemicals, and ROS), and the overexpression of exogenous tumor suppressors (p16INK4A and retinoblastoma (Rb)). The mechanism for this type of senescence involves the activation of CDKI, p16INK4A, and Rb [106].

CDKs mediate the transition between different phases of the cell cycle, and when interrupted due to DNA damage, the DDR is activated and the cell cycle is halted. Once halted, the cell has three fates: if DNA is repaired, the cell can re-enter the cell cycle; if the damage is too great to be repaired, the cell undergoes apoptosis and is cleared; and if the cell remains in cell cycle arrest, it can become senescent. Cell cycle arrest is the first step in the initiation of senescence and is characterized by an increased expression of many molecules, including cell cycle regulators like p16INK4A, p21 and p53, and senescence-associated β-galactosidase (SA-β-gal), which is only produced by senescent cells [154]. A more detailed description of vascular senescence, in particular in VMCs, can be found in our recent review [155].

It is now known that senescent cells are far from quiescent as they have a robust secretome called the SASP that develops later during full senescence. Components of this secretome include pro-inflammatory cytokines, such as IL-6 [156]; growth factors, including TGF-β [157]; proteases, such as MMPs; ECM components, such as fibronectin [158]; ROS [159]; and exosome-containing microRNAs [160].

The composition of the secretome depends on the cell type and inducer, as recently reported by Basisty et al. [161]. RAS overexpression, X-irradiation, and atazanavir, a drug used in cancer treatment, induced the expression of known markers, such as SA-β-gal, IL-6, and p16INK4A, as well as specific markers, depending on the cell type. For example, irradiation induced the expression of over 1500 proteins in fibroblasts and over 1100 proteins in epithelial cells. Importantly, many of the core components of the SASP coincide with aging markers identified in blood samples in humans, including growth differentiation factor 15 (GDF15), stanniocalcin 1 (STC1), the serine protease inhibitors SERPINs, and MMP-1. Both STC1 and MMP-1 have been associated with tumor growth and metastasis [162,163].

NOTCH1 is one of four NOTCH cell-surface receptors and is involved in a multitude of cellular processes, including fate determination, differentiation, proliferation, and death. Alternations of NOTCH1 signaling have been implicated in stress responses and can be tumor-suppressive or tumor-promoting. Its multitude of signaling and molecular targets was reviewed by Kopan and Ilagan [164]. Recently, Hoare et al. described NOTCH1 as a crucial regulator of unique SASP phenotypes in fibroblasts and ECs [165]. Protein assessment of oncogene-induced senescence (OIS) revealed that NOTCH1 activity during senescence is dynamic and can promote a balance between two SASPs. Specifically, NOTCH1 upregulates the TGF-β-induced secretome, inducing senescence in neighboring cells, while downregulating the C/EBPβ-associated expression of pro-inflammatory cytokines (IL-1, IL-6, and IL-8). Therefore, manipulation of NOTCH1 signaling can change the SASP composition without altering cell cycle arrest.

Senescent cells are upregulated in tissues during aging and senescence is believed to drive many age-related chronic conditions, including CVD, neurodegenerative disorders, and cancers. Since senescent cells are also known to increase with age, and the SASP drives inflammation, it is not a stretch that senescence plays a major, if not the largest, role in driving inflammation during aging [166].

### Cigarette Smoke and Nicotine in Senescence

Aging and smoking are two major risk factors in CVD development. Although recent reports have shown that smoking stimulates senescence in the lung [167,168,169], it is unknown whether smoking also accelerates senescence of the CV system. ECs isolated from atherosclerotic lesions in patients going through artery bypass graft surgery showed increased markers of inflammation, oxidative stress, and accelerated senescence, compared with non-smokers. Interestingly, smokers going through this procedure were an average of 10 years younger than non-smokers, suggestive of premature atherosclerosis induced by cigarette smoke [170]. The observation that senescence markers are upregulated by cigarette smoke in human plaques indicates that senescence could be relevant to vascular dysfunction induced by tobacco.

In VSMCs, senescence is induced by Ang II, which is the key effector of the rennin angiotensin system (RAS). Circulating Ang II induces hypertension, while the local and intracellular production of Ang II results in inflammation, cell proliferation, fibrosis [171], and atherosclerosis [172]. Ang II exerts its pathological effects by binding to AT_1_R, leading to Nox1 upregulation; ROS production; and phosphorylation of the kinases p38MAPK, Akt, and ERK [173,174]. Ang II also induces inflammation by activating the transcriptional factor NF-κB [175]. Once activated, NF-κB translocates to the nucleus, increasing the expression of proinflammatory cytokines, including IL-6, IL-1β, and TNF-α; chemokines, such as monocyte chemoattractant protein-1 (MCP-1); and cell adhesion molecules, such as vascular cell adhesion molecule-1 (VCAM-1) and intercellular adhesion molecule-1 (ICAM-1) [176]. An increased production of these pro-inflammatory molecules contributes to vascular injury and atherosclerosis. Furthermore, the binding of Ang II to AT_1_R induces vascular senescence in vivo [177] and in vitro in VSMCs [178].

Our previous work demonstrated that Ang II and zinc overload induce the senescence of VSMCs by an ERK-dependent mechanism [179] and that Nox1 mediates these effects [180,181]. Additionally, Nox4, an inducible enzyme that produces hydrogen peroxide, shows the opposite effects on senescence. In VSMCs, Nox4 downregulation induces senescence [182], while in ECs, reduced Nox4 expression inhibits senescence [183]. Nox2 downregulation was also associated with reduced EC senescence [184]. A more comprehensive review of the role of NADPH oxidases in vascular senescence can be found in our recent review [155].

Senescence is an ROS-dependent mechanism, so the upregulation of ROS generation systems like Nox1, or downregulation of antioxidant enzymes, such as catalase [179], or transcription regulators involved in ROS metabolism, such as FOXO1, the sirtuin Sirt1, or PGC-1α, which is a master regulator of mitochondria biogenesis, promotes senescence [185,186]. Our group also shed light on the mechanism by which PGC-1α modulates senescence by reporting that a reduced expression of sequestosome (SQSTM1/p62) in PGC-1α^−/−^ VSMCs mediates senescence by increasing ROS and reducing autophagy [187].

Nox1 increases ROS levels in response to tobacco smoke in VSMCs [142] and increases the expression of MMP-9 [188], which is also upregulated by nicotine. An increased expression of MMP-9 is associated with a poor prognosis in lung cancer [189] and breast cancer [190], and with increased inflammation associated with senescence [191]. Therefore, Ang II and nicotine share common signaling pathway cascades by activating p38MAPK and ERK signaling and inflammation, and by increasing the expression of Nox1 and MMPs. However, whether Nox1 also mediates cigarette smoke and nicotine effects in senescence remains to be elucidated.

## 5. Atherosclerosis Overview

The American Heart Association identifies well-known risk factors for atherosclerosis, including obesity, nicotine/tobacco use, drinking (alcohol), hypercholesteremia, hypertension, and type II diabetes [192]. Atherosclerosis is linked to physical inactivity and a Western diet, as well as genetics. The consumption of a Mediterranean diet is believed to protect the CV system by providing antioxidants (vitamins C and E) and polyphenols [193]. Antioxidants neutralize ROS produced during atherosclerosis, while polyphenols may increase the body’s endogenous antioxidant pool. We showed that polyphenols enriched in blackberry reduced the senescence of VSMCs by reducing Nox1 expression and upregulating the antioxidant response [181]. We also demonstrated that blackberry reduced plaque and Nox1 expression in a gender-dependent manner in ApoE^−/−^ mice [194].

Age and sex are other risk factors contributing to atherosclerosis. Risk is significantly increased for men at the age of 45 and women at the age of 55. In addition, a greater number of males have atherosclerosis and CVD, while estrogen is believed to be protective in females. Therefore, the decrease in estrogen levels that occurs during menopause heightens risk. Clinically, atherosclerosis is characterized by high amounts of total cholesterol (TC), low-density lipoprotein (LDL), and very low-density lipoprotein (VLDL) in the blood plasma. The major physiological role smoking plays in increasing the risk relates to the upregulation of LDL and heightened BP. In addition, the general inflammatory marker C-reactive protein (CRP) is often elevated and can predict one’s risk of adverse CV events. There are certain drugs that have been developed to treat atherosclerosis and CVD; however, none can prevent or completely reverse the diseases. Briefly, statins target hydroxymethylglutaryl coenzyme A (HMG-CoA) reductase and are very helpful in lowering cholesterol. Beta-blockers and angiotensin-converting enzyme (ACE)-inhibitors are also used, with the former targeting the RAS.

The initiation and progression of atherosclerosis begins with diffuse intimal thickening or fatty streak formation, which can occur at a very young age . The second stage is pathological intimal thickening, which occurs when more cholesterol and LDL enter the intima and macrophages and foam cells are present. This stage involves the migration of VSMCs to the intima. Lastly, advanced lesion formation occurs, in which a fibrous cap of VSMCs is developed, as well as a necrotic core with additional cholesterol, LDL, macrophages, and lipid-laden foam cells. During this step, calcification occurs [106].

### 5.1. Cell Types Involved in Atherosclerosis

The three major types of cells involved in atherosclerosis are ECs, VSMCs, and macrophages. During atherosclerosis initiation, oxidized lipids (oxLDL) and altered blood flow reduce eNOS, causing endothelial dysfunction, which involves the EC recruitment of circulating monocytes into the neointima. Platelet aggregation then occurs on the forming plaque cap and growth factors, such as PDGF, basic fibroblast growth factor (bFGF), and TGFβ recruits VSMCs to the neointima. Extracellular proteolysis is also required to break the basement membrane, allowing VSMCs to escape into the growing neointima. This process is mediated by MMPs, including MMP-3, MMP-9 [195], and MMP-2 [196]. A phenotypic switch from contractile to synthetic in VSMCs occurs and PDGFRβ, the receptor for PDGF, is upregulated, inducing VSMC migration to the neointima, where a small sub-set continues to proliferate. Ang II, acting through the membrane receptor AT_1_R, can lead to the hyperactivation of Akt and ERK signaling, driving VSMC migration and proliferation [197]. Recently, lineage tracing studies discovered that VSMCs can also differentiate into macrophages, evidenced by the concomitant expression of the contractile marker αSMA and macrophage marker CD68 in lesions [198]. In addition, calcium-containing osteochondrogenic cells (osteoblast and chondrocyte-like) with a VSMC lineage have been found in plaques [199]. VSMCs are stimulated by TGFβ, Ang II, PDGF, cholesterol, and IL-1, to produce large amounts of collagen in the fibrotic cap, thus altering the ECM volume and lesion composition [200,201].

VSMCs undergoing phenotypic switching secrete VEGF that influences the EC function [202] and secrete a variety of molecules that modulate plaque stability. Stable plaques have a strong fibrotic cap, characterized by a layer of VSMCs which secrete ECM components. Often, VSMCs become senescent, develop the SASP, and secrete IL-1α, which is the driver of the phenotype [10], as well as IL-6, IL-8, CCL2/MCP1, GROα/CXC1, G-CSF, FGF2, and PAI1, inducing plaque instability [203].

There are two fates for plaques—rupture and erosion—and different factors influence each outcome. A plaque more likely to erode is characterized by an abundance of the ECM component hyaluronan and the toll-like receptor 2 (TLR2) ligand that activates ECs, which in turn activate neutrophils [204,205]. Additionally, EC apoptosis induces platelet aggregation and thrombosis and eventually the plaque erodes [106]. On the other hand, plaques likely to rupture have heightened inflammation. They express IL-1α, IL-6, IL-8, and MCP1 [206]; have heightened NF-κB activity [207]; and MMPs expression (IL-1α drives MMP-2 and MMP-9 expression); and have an abundance of macrophages. IL-10 is also expressed, but confers protection, as it inhibits NF-κB activation and MMP expression [208]. Rupture is triggered by a weak and thin fibrous cap due to MMP-mediated ECM degradation and VSMC apoptosis. Rupture is a more common and serious outcome than erosion, as the larger plaque is more likely to cause a stroke or heart attack. While these events are not always fatal, they can cause irreversible damage, such as ischemia reperfusion injury [209].

### 5.2. VSMC Phenotypic Switch. in Atherosclerosis: The Role of MMPs

MMPs play an important role in atherosclerosis by promoting the VSMC synthetic phenotype. While MMPs are secreted by lymphocytes, macrophages, ECs, and VSMCs, macrophages of a monocyte or VSMC origin are believed to be the most significant producer of MMPs. A low-fat diet alone or in combination with statins can significantly reduce macrophages and MMPs [210,211]. There are 25 different types of MMPs, including membrane-bound and non-membrane-bound MMPs that can degrade distinct components of the ECM. While matrix degradation was previously believed to be their primary function, it is now known that certain MMPs play a role in cell signaling cascades. Namely, MMPs mediate the cleavage of ECM-bound cytokines and growth factors [212].

MMPs are grouped into categories based on their targets. These groups include collagenases (MMP-1, -8, and -13), gelatinases (MMP-2 and -9), stromelysins (MMP-3 and -10), matrilysins (MMP-7 and -26), and membrane type (MT-MMPs). Löffek et al. have provided detailed information on the MMP structure and function [212]. Importantly, MMPs are synthetized as inactive proenzymes, and can thus be regulated by pro-peptide activation. They are also regulated at the transcriptional level and by endogenous tissue inhibitors (TIMPs).

Certain MMPs and TIMPs are present in the arteries in basal amounts. MMP-2, TIMP1, and TIMP2 are three that are constitutively produced by both arterial ECs and VSMCs [213]. However, although they are expressed, they are not active. In diseased arteries, certain MMPs are active. Two of the most widely studied in the context of vascular disease are MMP-2 and MMP-9 [195,196,214].

In atherosclerotic specimens from human patients, MMP expression correlates with the stability of the fibrous cap. Sluijter et al. measured MMP-2, MMP-8, and MMP-9 expression and found that the former was more abundant in VSMC-rich fibrotic lesions, while the latter two were more abundant in macrophage-rich inflammatory lesions. Therefore, they concluded that MMP-2 was associated with a more stable plaque, while MMP-8 and -9 correlated with a vulnerable and unstable plaque [215]. Additionally, in human-derived atherosclerotic tissue samples, Choudhary et al. assessed MMP-2, MMP-9, and TIMPs, and found that all MMPs were more abundant in the plaque area, most significantly, MMP-9. On the other hand, MMP-2 was the most abundant in normal, non-plaque areas. Lastly, TIMPs were present in higher amounts in the fibrotic and necrotic portions of the plaque [216]. Overall, the expression of distinct MMPs in different sections of the plaque contributes to the differential plaque composition and stability.

Signaling cascades influencing the VSMC phenotypic switch can be induced by MMPs. For example, MMP-6 can induce p38MAPK signaling in VSMCs. Specially, this MMP induces the specific p38MAPK isoform MAPK14, which is expressed at high levels in VSMCs and human saphenous veins (HSV) upon injury, compared to other family members. Wu et al. [217] overexpressed MAPK14 in mouse VSMCs and observed a lower expression of contractile genes, including MYHC. Additionally, ligation injury of the MAPK14 smooth muscle specific knockout (iSMC-MAPK14^−/−^) model showed substantially reduced neointima formation and a reduced expression of pro-inflammatory molecules.

### 5.3. Nicotine Interaction with Angiotensin II

RAS is important in systemic homeostasis as it regulates the electrolyte balance and BP. Hyperactivation of this system can lead to hypertension, stressing the heart and vasculature and contributing to the development and progression of atherosclerosis [218]. Ang II is a major player in this system and controls the vascular tone through AT_1_R receptors. Ang II induces the G-protein (G_q_)-mediated activation of phospholipase C (PLC) and inositol triphosphate (IP_3_) production, which increases intracellular Ca^2+^, activating MLCK, which phosphorylates MLC, leading to VSMC contraction. G_q_ signaling can also activate Janus kinase 2 (JAK2) and ROS, causing an increase in intracellular Ca^2+^ and MLCK-mediated phosphorylation of MLC. In addition, other molecules, such as calmodulin protein kinase II (CaMKII) and c-Src, can phosphorylate MLC, resulting in vasoconstriction [218].

Groundbreaking research characterizing the systemic RAS occurred in the 1800s up to the mid-1900s [219,220,221]. Recently, it has been discovered that there are also local RAS systems. For example, the vasculature can produce all components of the RAS except renin, which enters the system from circulating blood. Local vascular RAS affects VSMCs, inducing differentiation, proliferation, migration, and apoptosis. Overall, the actions of RAS in the vasculature facilitate tissue remodeling. Furthermore, hyperactive vascular tissue RAS can lead to pathological states, such as atherosclerosis [222].

There are two Ang II receptors: AT_1_R and AT_2_R. Generally, most of the pathophysiology occurs via AT_1_R. Both systemic and local RAS contribute to vascular stiffness when Ang II acts through the AT_1_R receptor. Action through the AT_1_R can transactivate ADAM metalloproteinase domain 17 (ADAM17) and EGFR, leading to Akt and ERK signaling cascades, and upregulate the transcription of hypertrophy genes. In addition, ERK, as well as c-Src, c-Jun, and β-Catenin downstream of AT_1_R, result in hypertrophic and proliferative activity. ROS production via Nox and AT_1_R activity can also lead to vascular hypertrophy and hyperplasia and endogenous intracellular antioxidants, such as SOD1, can ablate ROS activity [218].

Ang II actions exacerbate the natural process of aging, which involves arterial remodeling and stiffening of the arteries. Ang II leads to vascular remodeling, atherosclerotic neointima formation, and arterial stiffness through the regulation of MMPs and their endogenous inhibitors. The main players MMPs and TIMPs must remain in balance so that MMP-induced VSMC proliferation and migration and neointima formation does not occur [218]. In addition, Ang II plays a role in inflammation processes in vessel walls. Ang II activates NF-kB, driving cytokine production and inflammation. Ang II signaling is also involved in aging, as AT_1_R inhibition in mice can prolong longevity [223].

Nicotine exacerbates the effects of Ang II by increasing MMP-2 expression and vascular hypertrophy that promotes a stronger hypertensive response, compared with Ang II alone [224]. Importantly, maternal nicotine exposure exacerbated hypertension and vascular contractility in responses to Ang II in the offspring [225,226]. Furthermore, a blockade of AT_1_R was shown to reduce cardiac remodeling and ischemia reperfusion in response to nicotine [227]. Additionally, a review of the interaction between nicotine and RAS by Oakes et al. [228] revealed that nicotine upregulates the pathological ACE/AT_1_R axis, while reducing the protective ACE2/AT_2_R axis of RAS.

### 5.4. Tobacco and Nicotine in Atherosclerosis: Cell and Animal Models

While both the ApoE^−/−^ and LDLR^−/−^ murine models are well-known and widely used to study atherosclerosis due to hypercholesterolemia [229,230] and have been used to test possible therapeutic interventions, the former is better suited for tobacco and nicotine studies. The ApoE^−/−^ mouse model is set-apart from the LDLR^−/−^ mouse model because the lack of endogenous ApoE results in severe cholesterol accumulation in macrophages, which triggers an inflammatory response and breakdown of the ECM by cytokine and protease secretions, respectively. The LDLR^−/−^ is not characterized by such inflammatory and matrix degradative responses. The ApoE^−/−^ model is used to study other diseases in which severe inflammation and ECM degradation occur, such as Alzheimer’s [231]. The ApoE^−/−^ model can also be useful for studying the smoking-exacerbated condition COPD, in addition to atherosclerosis, in tobacco and nicotine studies. In fact, along with developing early-onset atherosclerosis, ApoE^−/−^ mice develop emphysema due to the impaired development of lung alveoli [232]. The development of both atherosclerosis and lung emphysema can be hastened by feeding animals a Western diet or high-fat diet (HFD) in place of a normal chow diet [233].

A PubMed search (March 14, 2020) of “nicotine AND ApoE” yielded only 41 results, while a search of “cigarette smoke AND ApoE AND atherosclerosis” yielded 46 results. There is a large overlap in the hits between these two searches. Generally, older studies exclusively use cigarette smoke, while several newer studies investigate how E-cigarettes and their vapors affect cardiorespiratory systems. Of note, not all the studies using ApoE^−/−^ mice determined atherosclerotic plaques. For example, Tani et al. exposed 20-week-old ApoE^−/−^ on a Western diet to 5 weeks of cigarette smoke, followed by carotid arterial cuffing, and found increased intimal thickening [234]. No plaque was reported in this study. These searches revealed the paucity of research in this important area, although more research is being conducted in light of the recent mortality associated with the use of vaping systems.

### 5.5. Cigarette Smoke

Previous studies in the ApoE^−/−^ mouse model have demonstrated the adverse effects that cigarette smoke has on the CV system, and how cessation can mitigate or reverse these negative outcomes. These studies have been summarized by Lo Sasso et al. [233]. For example, Gairola and colleagues tested whether sidestream smoke interfered with the common smoking cessation aid varenicline. In ApoE^−/−^ mice fed a Western diet, they found that long-term sidestream smoke exposure significantly increased the plaque area in the aorta and esterified and unesterified cholesterol accumulation in aortic tissues [235]. Similarly, Knight-Lozano et al. observed an increased plaque in response to 3–6 weeks of cigarette smoke exposure 5 days per week in male ApoE^−/−^ [236]. This effect was also associated with a reduced expression of mitochondrial enzymes, including SOD2 and overall mitochondrial dysfunction. A study by Matetzky et al. analyzed the plaque composition in male ApoE^−/−^ mice on a high cholesterol diet exposed to cigarette smoke 5 days a week for 8 weeks [237]. The study showed that the plaque in the smoking group had higher levels of tissue factor (TF), which is a molecule involved in thrombus formation, suggesting that cigarette smoke may promote a plaque prone to rupture. A change in the atherogenic lipidome was also reported using ApoE^−/−^ mice exposed to long-term mainstream cigarette smoke 5 days a week [238]. Cigarette smoke increased different types of lipids, including free cholesterol, ceramides, and phospholipids, in the plasma; free and esterified cholesterol in the liver; and cholesterol ester and lysophosphatidylcholine in the aorta. Interestingly, Kunitomo et al. demonstrated that the exposure of ApoE^−/−^ mice to the gas phase of smoke (lacking nicotine and tar) was sufficient to induce oxidative stress in blood circulation (oxLDL) and in the aorta (3-nitrotyrosine), while increasing the TC [239]. These data suggest that other components in cigarette smoke, in addition to nicotine, also contribute to the development of CVD.

### 5.6. Nicotine Vapors

Recent studies have investigated the effect of nicotine vapors in atherosclerotic animal models, with many concluding that they are safe alternatives to cigarette smoke. While the 3R4F cigarette is commonly used, as a reference cigarette, in smoking animal studies, there is not a standard e-cigarette juice used to assess the harm of these products in animal studies, so the composition of the vapors is variable. A major consideration is also the source of funding. Two very recent studies were funded by Philips Morris International (PMI) [73,240]. Historically, PMI is well-known for selling Marlboro tobacco products, but now claims to be “building a smoke-free future” by delivering smoke-free alternative products to consumers. While PMI has its headquarters in the USA, it is an international company [241]. PMI was operating under Altria until 2008, when the latter company decided to focus solely on USA markets. Shareholders in Altria were given shares in PMI and PMI, rebranded as Phillips Morris USA in America, and Altria still owns these brands. In 2018, Altria bought 35% of ownership of JUUL—a leading electronic cigarette brand [242,243]. Pisinger et al. have described the conflict of interest involved when companies with profit to gain fund research on nicotine vapors. Using Fischer’s Test and Logistic regression, Pisinger et al. observed that studies with a stake in the market, on average, found harm 39% of the time, while studies without a financial interest found adversity 95% of the time [244].

The first study analyzing a cigarette alternative by PMI in ApoE^−/−^ mice was published in 2016 [74]. It was an 8-month long study in female mice comparing cigarette smoke to their tobacco aerosol delivering device, which they called a Modified Risk Tobacco Product (MRTP), Tobacco Heating System THS 2.2. MRTPs strive to be, as the name suggests, a better alternative to smoking traditional cigarettes. The philosophy behind THS 2.2 and other similar aerosol systems, is a heat-not-burn philosophy, i.e., the vapor is heated not burned [240]. Heated tobacco vapors are designed to negate harmful combustion products. It is well-known that the harmful combustion products of cigarettes are implicated in a heightened disease risk and disease endpoints. However, the FDA warns against all tobacco use, stating that there is no safe tobacco product, and that the nicotine in these products makes them highly addictive. Additionally, other tobacco constituents are known carcinogens, which thus increase one’s risk for cancer [245]. Collectively, the harmful constituents of burned tobacco are referred to as Harmful and Potentially Harmful Constituents in Tobacco Products (HPHCs) by the FDA. In Fall of 2019, the FDA published a notice suggesting that 19 e-cigarette toxicants, including the e-liquid heated byproduct glycidol from glycerol, be added to the HPHCs list [246].

Despite FDA’s clear warnings about the harm of all tobacco product use, the initial and subsequent study by PMI in ApoE^−/−^ mice tested both THS 2.2 and another MRTP e-cigarette model CHTP 1.2 with equal nicotine levels [74,240]. In comparison to 3R4F, the study concluded that their devices were significantly less damaging to the CV system than cigarette smoke. Therefore, they recommend these devices as safe alternatives to cigarette smoke.

Another recent study by Szostak et al. also found that e-cigarette aerosols were less harmful to the CV system than the smoke from cigarettes [73]. This study included five groups of ApoE^−/−^ mice—control, test cigarette, carrier aerosol, nicotine-containing base aerosol, and test vape aerosol with nicotine and flavoring. The smoke and aerosols were delivered for the same duration and time span as Phillips, 2019, and delivered equal nicotine to groups [240]. The dosage of nicotine delivered to the mice was 193 µg per day, which corresponds to a dose of 37.5 mg/day in humans. The study duration was chosen in order to assess lung and CV progression accurately and correspond to human use. Specifically, 6 months represents about a quarter of a mice’s lifespan and 20 years for a human. Females were chosen in all three of these studies because, in mammals, females are more at risk for smoke and possibly vape-related diseases such as COPD, as well as atherosclerosis, although the literature is inconclusive [73,74,240].

However, a study conducted in an academic setting had very different findings. They used a whole-body chamber system to deliver cigarette smoke in 0% and 2.4% nicotine doses to male ApoE^−/−^ mice. They found that nicotine caused impaired, ventricular systolic, but not diastolic, function in the heart. In addition, ROS, mitochondrial DNA damage, and plaque in the aortic root were significantly higher [247].

### 5.7. Additional Nicotine Studies

A number of studies have also been conducted to elucidate the specific role of the tobacco constituent nicotine in VSMCs and the progression of atherosclerosis. The gold standard design of these studies uses cultured cells to define the mechanism and ApoE^−/−^ mice to assess disease progression, and generally, these studies are conducted in a university setting. Table 1 describes atherosclerotic studies that have employed the ApoE^−/−^ model.

Mechanistic studies have observed that nicotine exerts effects via the α1-nAChR. Ren et al. [248] used cell culture and an ApoE^−/−^ model to observe changes induced by nicotine in the α1-nAChR. Nicotine induced changes via this receptor to further the progression of atherosclerosis by increasing LDL, triglyceride (TG), and TC, and reducing high-density lipoprotein (HDL). In addition, nicotine upregulated IL-6, TNF- α, and IL-10, as well as atherosclerotic-associated markers calpain-1, MMP-2, and MMP-9. Knockout of the receptor conferred protection, observed by decreased plaque development in vivo. Xu and colleagues [249] demonstrated that nicotine acts in VSMCs through nAchRα1 to phosphorylate STAT3, Akt, and mammalian target of rapamycin (mTOR), upregulating MMP-2 and increasing cell migration and proliferation. They also observed that nicotine increased inflammation in macrophages by the upregulation of MCP-1 and the pro-inflammatory factor IFN-γ and by downregulation of the anti-inflammatory factor IL-10. The study used an ApoE^−/−^ mouse model fed a HFD, nicotine in drinking water, and a knockout of nAchRα1. The results indicated that the knockdown of nAchRα1 reduced p-STAT3 and was able to attenuate plaque development induced by nicotine. Therefore, this is a major pathway leading to atherosclerotic lesion formation [249].

A recent study using mouse VSMCs (MASMs) and ApoE^−/−^ mice demonstrated that nicotine activated ROS-dependent NF-κB signaling in VSMCs to regulate autophagy, indicated by higher LC3 I to LC3 II conversion and decreased SQSTM1/p62 expression. The inhibition of autophagy in nicotine-treated cells prevented the VSMC phenotypic switch and upregulation of the ECM-remodeling protein osteopontin, VSMC migration, and collagen I synthesis. Nicotine-treated cells had higher ROS levels and antioxidant treatment reduced ROS and prevented the phenotypic switching by reducing autophagy and inhibiting collagen formation. In corroboration with other studies, HFD and nicotine treatment significantly increased the aortic lesion area compared to HFD alone. The importance of this study is that inhibiting autophagy can reduce VSMC proliferation and migration, thus reducing plaque formation. Furthermore, while in the early stages of atherosclerosis development, autophagy may be beneficial to clear cellular debris and protect against cellular distress and oxidative stress, during the later states, abnormal autophagy (severely high or unregulated) may be harmful, leading to the death of cells in the cap, and increasing plaque instability and the risk of plaque rupture [250].

The role that macrophages play in response to nicotine in atherosclerosis can also be observed in ApoE^−/−^ mice. Zhu et al. investigated the role that macrophages have in the acceleration of atherosclerosis using RAW264.7 and MASMs in vitro and ApoE^−/−^ mice in vivo. Specifically, they investigated the role of exosomes, which are secreted by macrophages and regulate VSMCs in atherosclerosis. They found that macrophages treated with nicotine secrete exosomes with miR-21-3p, which targets phosphatase and tensin homolog (PTEN), an Akt phosphatase, to induce VSMC migration and proliferation and further plaque development [251]. Interestingly, varenicline, known as one of the best drugs in smoking cessation, aggravated plaque accumulation in ApoE^−/−^ mice treated with nicotine by upregulated uptake of oxLDL in macrophages [252], suggesting that people going through smoking cessation therapy may still be at risk of developing and/or worsening CVD.

Another study examined the role that mast cells (MCs) have in nicotine-accelerated atherosclerosis. The results indicated that nicotine acts through α7 nAChRs on MCs to exacerbate atherosclerosis by enhancing lesion development; a process that can be blunted by knocking the cells out in vivo [253].

Studies examining the role of ECs have also been conducted in ApoE^−/−^ mice. Li et al. found that nicotine in human umbilical vein endothelial cells (HUVECs) and ApoE^−/−^ mice caused endothelial dysfunction and promoted atherosclerosis. They found that the overexpression of GTP cyclohydrolase 1 (GTPCH1), the rate-limiting enzyme in tetrahydrobiopterin (BH4) de novo synthesis, and the BH4 supplement were protective against nicotine-accelerated atherosclerosis [254]. Wu and colleagues used human aortic ECs (HAECs) and ApoE^−/−^ to assess changes in EC pyroptosis or cell death mediated by the inflammasome and caspase-1 activity. In nicotine-treated ECs, ROS activated pyroptosis characterized by the activation of NACHT, LRR and PYD domain-containing protein 3 (NLRP3) inflammasome, caspase-1, and interleukin (IL-1β and IL-18) production, which was inhibited by the ROS scavenger N-acetyl-cysteine (NAC). In vivo, nicotine increased the plaque area and lipid content, suggesting that activation of the inflammasome may promote disease progression in vivo [255].

The mechanism of action of cigarette smoke and nicotine in other pathological vascular disorders, such as abdominal aortic aneurysms (AAA), has also been investigated in the ApoE^−/−^ model. Stolle et al. [256] demonstrated that cigarette smoke exacerbated Ang II-induced AAA formation, which was associated with an increased expression of MMP-2, -3, -8, -9, and -12. Wang et al. [253] showed that nicotine also exacerbated Ang II-induced AAA formation in ApoE^−/−^, but not in ApoE^−/−^ AMPK-α2^−/−^ double knockout. In VSMCs in culture, nicotine and Ang II activated AMPK-α2, leading to MMP-2 expression.

## 6. Conclusions

A plethora of evidence has shown that cigarette smoke is a major contributor to atherosclerosis. However, the role of nicotine and nicotine metabolites, such as cotinine, requires further investigation. This is relevant, as nicotine vaporization products have become more popular in recent years. Nicotine appears to increase oxidative stress and the inflammatory burden and accelerate atherosclerosis to increase the risk for CVD. However, it is unclear whether nicotine and cotinine affect VSMC phenotypic switching to a proliferative, migratory phenotype and atherosclerosis development and VSMC senescence, characteristic of a more advanced plaque. The summation of these events is relevant in the context of atherosclerosis severity as they promote an unstable atherosclerotic cap prone to rupture. A ruptured plaque may block an artery and lead to ischemia, restricted tissue blood flow, which impairs tissue oxygenation and pathological endpoints, such as a heart attack or stroke, by blocking arteries in the heart or brain.

## Figures and Tables

**Table 1:** Tobacco and nicotine in atherosclerosis: cell and animal models.

Ref	Cell/ Mouse Model	Experimental Design	Dose and Treatment Duration	Major Findings	Funding Source
[74]	ApoE^−/−^ females 8–10 w old	CS (3R4F) THS 2.2 Cessation (2 m CS + 6 m C) Switch (2 m CS + 6 m THS 2.2)	29.9 mg/m^3^ CS whole-body exposure 3 h/d, 5 d/w for 8 m	CS significantly altered lipid profiles, increased the plaque area, and induced lung inflammation and emphysema, compared to aerosol. Cessation or switching from CS to aerosol reversed the disease outcome.	PMI
[240]	ApoE^−/−^ females 8–10 w old	CS (3R4F) CHTP 1.2 THS 2.2 Cessation (3 m CS + 3 m C) Switch (3 m CS + CHTP 1.2)	28 µg N/L whole-body exposure 3 h/d, 5 d/w for 6 m	CS increased the plaque, inflammation, and emphysema, compared with aerosol. Cessation and switch to aerosols reduced negative outcomes induced by CS to near sham.	PMI
[73]	ApoE^−/−^ females 12–14 w old	PG, VG PG, VG, N PG, VG, N, flavor CS (3R4F)	35 µg N/L (4%) whole-body exposure 3 h/d, 5 d/w, 6 m	CS compared to aerosols and C had more plaque in the aorta, ventricle dysfunction, and increased plasma lipids.	PMI
[247]	ApoE^−/−^ males 8 w old	Western diet: Saline aerosol Tobacco w/o N aerosol Tobacco+N aerosol	2.4% N whole-body exposure 12 w	E-cigarette with N, but not without N, impairs the ventricular systolic function in the heart, increases ROS and mtDNA damage, and increases the amount of plaque in the aortic root.	NIH DPIDARP CTRDRP NIMHHD
[248]	MASM RAW264.7 ApoE^−/−^ males 8 w old	N HFD and N AAV+N, α1-AAV+N	0.5 ng/mL I.P. 2.0 mg/kg/day, 12 w	α1-AAV with N had less plaque and a different lesion content (reduced collagen and lipids). N via α1-nAChR altered the lipid profile and induced inflammation and MMPs. N’s atherosclerotic effect involved calpain-1/MMP2/MMP9 signaling.	NNSF of China
[249]	MASM RAW264.7 ApoE^−/−^ males 8 w old	N HFD and N N+AG490 (STAT3 inhibitor), AAV+N, α1-AAV+N	100, 500 ng/mL N in drinking water (100 µg/mL), I.P. AG490 (100 µg), Virus once − 1 × 10^11^/mL (200 μL), 12 w	N activates STAT3 via nAchRα1. Knockdown of nAchRα1 or STAT3 inhibition reduces N’s effect on SMC proliferation and migration and macrophage inflammation by blunting Akt/mTOR/MMP2 signaling. In vivo, STAT3 inhibition decreases N-induced lesion development.	NNSF of China
[250]	MASM ApoE^−/−^ 8 w old	N C HFD HFD+N	10 µM N, 36 h, 10 mM 3-MA, 100 nM rapamycin 2 mg/kg/d, 12 w	N exacerbates plaque formation. Targeting the N-induced nAChRs/ROS/NF-κB pathway can reduce autophagy and decrease VSMC phenotypic switching to reduce plaque formation.	NNSF of China CTCCR
[251]	MASM RAW264.7 ApoE^−/−^ males 8 w old	N HFD HFD+N	10^−5^ M, 24 h PBS or S.C., 2 mg/kg/d 12 w	N accelerates plaque formation. Macrophages in the plaque secrete exosomes with miR-21-3p, which increases VSMC migration and proliferation in a PTEN-dependent manner.	NNSF of China
[254]	HUVEC ApoE^−/−^ males 8–12 w old	N HFD with or w/o N and BH4 HFD with or w/o N and LV.GFP or LV.GTPCH1	1 µmol/L, 48 h 100 mg/L N in drinking water 10 mg/kg BH4/d 12 w	In vitro, N inhibited GTPCH1, reduced NO, and increased ROS. In vivo, N caused endothelial dysfunction, and increased ROS and the plaque area, while GTPCH1 overexpression or BH4 supplementation attenuated N’s effects.	NNSF of China
[255]	HAECs ApoE^−/−^ 8 w old Gender ND	N	1 µM N 24 h 100 µg/mL in drinking water 12 w	Nicotine increased the ROS-induced inflammasome NLRP3-ASC and pyroptosis in HAECs. With both diets, N increased the plaque lipid content and area.	NNSF of China CRG NCET
		ND			
		ND+N			
		HFD			
		HFD+N			
[253]	ApoE^−/−^	HFD and N, MC^−/−^ and α7nAChRs^−/−^+N	100 µg/mL in drinking water, 12 w	Nicotine activates α7 nAChRs on MCs to enhance atherosclerosis. N and HFD developed lesions, which were diminished in MC^−/−^ mice.	NNSF of China NBPR of China, etc.
[239]	ApoE^−/−^ 5 w old	HFD diet: Smoke Smoke+Vit. E	Gas phase smoke whole-body exposure, 15 mi/d, 6 d/w, 16 w	Smoke increased oxLDL and cholesterol in plaque and 3-nitrotyrosine in mouse aortas. Vit. E reduced smoke-induced effects.	SRF OPCPMWU
[252]	RAW264.7 ApoE^−/−^ 8 w old	HFD 5 w	Varenicline 0.5 mg/Kg/d S.C. 300 µg/mL N in drinking water	The smoking cessation aid varenicline upregulated N-induced oxLDL uptake in macrophages, decreasing cholesterol efflux and exacerbating atherosclerotic.	GASR CRIF, Japan
[235]	ApoE^−/−^ Females 8–9 w old	Western diet	Sidestream CS (25 ± 2 mg/m^3^) whole-body exposure, 6 h/d, 5 d/w 7, 10, 14 w	Sidestream CS exposure significantly increased the aortic plaque area and cholesterol in aortic tissues of atherosclerotic mice.	Gill Heart Institute, US
[236]	ApoE^−/−^ Males 7 w old	HFD	Sidestream CS (30 mg/m^3^) whole-body exposure, 21–41 d (6 h/d, 5 d/w)	Secondhand smoke in combination with hypercholesteremia increased lesions and mitochondrial damage.	NIH
					CTRDRP
					CFR
					JSF

3-MA: 3-methyladenenine, AAV: control shRNA, α1-AAV: nAChRα1 shRNA, CS: cigarette smoke, S.C.: subcutaneous injection, C: control, CTRDRP: California Tobacco-Related Disease Research Program, CHTP 1.2: Carbon Heated Tobacco Product by PMI delivering nicotine aerosol, CRG: Creative Research Groups, CFR: Clayton Foundation for Research, CRIF: Central Research Institute of Fukuoka, CTCCR: Clinical Research Innovation Plan of Shanghai General Hospital, d: day, DPIDARP: Diversity-Promoting Institution Drug Abuse Research Program, GASR: Grant-in-Aid for Scientific Research, h: hour, HFD: high-fat diet, I.P.: intraperitoneal injection, JSF: John Sealy Foundation, L: liter, m: minute, MC: mast cells, m: months, NBRP: National Basic Research Program of China, NIMHHD: National Institute on Minority Health and Health Disparities, N: nicotine, ND: normal diet, NCET: New Century Excellent Talents In Heilongjiang Provincial University, NNSF: National Natural Science Foundation of China, OPCPMWU: Open Research Center Project of Mukogawa Women’s University, PG: propylene glycol, PMI: Philip Morris International, THS 2.2: Tobacco Heating System by PMI delivering nicotine aerosol, SRF: Smoking, VG: vegetable glycerol Research Foundation, w: weeks.
